# Prevalence and risk factors for dental caries among 3-year-old children in Shanghai, China: a cross-sectional study

**DOI:** 10.1186/s12903-025-06454-9

**Published:** 2025-07-09

**Authors:** Shuran Yao, Jing Zhu, Hao Zhang, Huning Wang, Dongxin Da, Jin Yu, Yiwei Jiang, Hongru Su, Hongyan Shi, Qiwen Chen, Zhengang Wu, Jiangtao You, Xiaoli Zeng, Ying Zhang

**Affiliations:** 1 Department of Preventive Dentistry, Shanghai Stomatological Hospital, School of Stomatology, Fudan University, Shanghai, China; 2 Shanghai Key Laboratory of Craniomaxillofacial Development and Diseases, Fudan University, Shanghai, China; 3 Xuhui District Dental Centre, Shanghai, China; 4 Minhang District Dental Centre, Shanghai, China; 5 Jing’an District Dental Centre, Shanghai, China; 6 Pudong District Dental Centre, Shanghai, China; 7 Putuo District Dental Centre, Shanghai, China

**Keywords:** Caries incidence, Severe early childhood caries, Dental caries, Primary teeth, China

## Abstract

**Background:**

Early childhood caries (ECC), especially severe early childhood caries (SECC), severely affects children’s oral health, causing pain and tooth loss. This study aimed to investigate the prevalence of dental caries among 3-year-old children in Shanghai, China, and to identify associated risk factors.

**Methods:**

This cross-sectional study involved 1,241 3-year-old children from six Shanghai districts, selected via stratified random sampling. The study used an oral health questionnaire and clinical examinations by trained examiners following WHO guidelines. Dental caries were diagnosed using WHO criteria, with dmft scores indicating caries presence (≥ 1) and SECC defined as ≥ 4. Caregiver-completed questionnaires gathered data on oral hygiene practices and sociodemographic information. Statistical analyses included the Mann-Whitney U test, Kruskal-Wallis H test, chi-square test, logistic regression, and zero-inflated negative binomial (ZINB) regression.

**Results:**

The prevalence of dental caries among 1241 children was 25.00%, with a mean dmft score of 0.98. The prevalence of SECC was 9.43%. The significant caries index (SiC) and SiC10 were 2.94 and 7.02, respectively. Logistic regression analysis indicated that consuming sugar-sweetened beverages (OR = 1.65, 95% CI 1.07–2.55, *p* < 0.05), eating after bedtime brushing (OR = 1.62, 95% CI 1.01–2.59, *p* < 0.05), the age at which tooth brushing commenced (OR = 2.63, 95% CI 1.40–4.96, *p* < 0.05), and the mother’s education level (OR = 1.61, 95% CI 1.07–2.43, *p* < 0.05) were associated with SECC occurrence. ZINB regression analysis revealed that consuming sugar-sweetened beverages (OR = 0.45, 95% CI 0.15–0.74, *p* < 0.05) and eating after bedtime brushing (OR = 0.67, 95% CI 0.34–1.01, *p* < 0.001) were associated with the incidence of dental caries, whereas the age at which tooth brushing commenced (IRR = 1.58, 95% CI 1.13–2.20, *p* < 0.05) and the mother’s education level (IRR = 1.47, 95% CI 1.15–1.87, *p* < 0.05) were related to the dmft score.

**Conclusions:**

The prevalence of dental caries among 3-year-old children in Shanghai is low, yet the rate of SECC remains a concern. SECC is associated with factors including the frequency of sugar-sweetened beverage intake, post-toothbrushing eating habits, age of toothbrushing initiation, and mother’s education level.

## Background

Dental caries is one of the most common chronic diseases and is a serious public health problem caused by interactions among cariogenic bacteria, carbohydrates, improper nutritional conditions, and some social factors affecting people of all age groups and socioeconomic backgrounds [1–3]. The prevalence of dental caries is greater in developing countries than in developed countries and Asia than in Europe and Latin America [2, 4]. According to the 2022 Global Oral Health Status Report, it is estimated that 514 million children worldwide have caries in their deciduous teeth [5].

Early childhood caries (ECC) refers to the presence of one or more decayed, missing (due to caries), or filled tooth surfaces in any tooth of a child under 72 months of age (6 years old). Severe early childhood caries (SECC) can be diagnosed if a child under 3 years of age has smooth surface caries, or if a 3-year-old child has a decayed, missing, or filled tooth (dmft) score of ≥ 4, or if a 4-year-old child has a dmft score of ≥ 5, or if a 5-year-old child has a dmft score of ≥ 6 [6]. Worldwide, data show that ECC remains very common; although it is highly preventable, it is still largely untreated in most cases [7]. The global prevalence of ECC is 48% [8].

ECC has a negative impact on children’s well-being, learning skills, growth, and development. ECC can lead to premature tooth loss, malnutrition, and delayed growth and development and can reduce children’s oral health-related quality of life [9–11]. Owing to the high cost of advanced SECC treatment under general anaesthesia or sedation, which many families cannot afford, SECC also imposes a heavy economic burden on families [4]. In addition, the risk of caries in deciduous teeth increases with age [12, 13], especially for children who have already had caries; the risk of caries is even greater [14]. A 2021 systematic review identified “age” as a variable in the Caries Risk Assessment (CRA) tool because of its consistent predictive value for future caries risk. A study revealed that 3-year-old children who already had caries experienced greater increases over the next 30 months than did caries-free children [14]. Children with caries in their deciduous teeth are more likely to have caries in their permanent teeth [15]. Therefore, interventions should target the oral health behaviors of children aged 3 years and younger to mitigate the impact of the SECC.

A systematic evaluation of the prevalence of dental caries among Chinese residents from 1980 to 2018 revealed that the prevalence among residents in mainland China was generally high and has been increasing over the past 38 years. Regional disparities still exist between the eastern and western parts of China and rural and urban areas [16]. As an international metropolis, Shanghai has topped the list of gross domestic products (GDPs) among cities in China for several consecutive years. From 2015 to 2021, Shanghai’s economy continued to grow, and its global influence increased daily. In 2021, the GDP of Shanghai reached the level of upper-middle developed countries. However, the public’s attention to children’s oral health significantly differs from that in developed countries and regions. The fourth national oral health epidemiological survey in 2015 revealed that the caries situation among children in mainland China was severe, with a caries prevalence of 50.8% among 3-year-old children, 63.6% among 4-year-old children, and 71.9% among 5-year-old children [17]. The caries prevalence among 3-year-old children in Shanghai is 45.7% [18], which is lower than the national average of 50.8% [17] but higher than the caries prevalence of 38% among 3-year-old children in Hong Kong in 2016 [19]. ECC is closely related to microorganisms, genetic factors, dietary habits, behavioural habits, mothers’ education level, family socioeconomic status, and medical level [20–22]. However, there are significant differences in the composition and degree of cariogenic factors, such as regional and social background, under different conditions.

Since the last national oral health survey in 2015, no updated data on the prevalence and risk factors for dental caries among 3-year-old children in Shanghai have been available. This has limited the assessment of the impact of the “Comprehensive Intervention Programme for Children’s Oral Diseases” initiated in response to the high caries prevalence identified in 2015. The programme primarily focuses on providing oral health education to parents and caregivers, promoting regular dental check-ups, and applying fluoride varnish in some regions. To comprehensively assess the oral health status, behavioural factors, and health literacy of urban and rural residents in China and understand their trends over time, a nationwide oral health surveillance programme for key populations was initiated in 2021. The data for this study were derived from the oral health surveillance conducted in Shanghai in 2021, aimed at updating the prevalence and risk factors for dental caries among 3-year-old children in Shanghai since 2015. This study aims to analyse the caries status of 3-year-old children in Shanghai and related risk factors, thereby providing essential evidence for the development of targeted oral prevention policies and strategies in Shanghai.

## Methods

### Study design

This study draws upon data from the 2021 China Chronic Disease and Nutrition Surveillance Project and explicitly focuses on the oral health status of key populations in Shanghai. Six districts, comprising four urban districts and two suburban districts, were selected for this study. The selection adhered to cluster sampling principles, employing a kindergarten-based sampling method. Specifically, four kindergartens were chosen from each monitoring site as survey units, and 56 three-year-old children were randomly selected from each kindergarten to serve as research subjects.

### Study participants

Children who did not comply with the dental examination procedures or were absent on the examination day were excluded from the study. The age of the participants was determined by calculating the difference between their date of birth and the examination date, and only children who were precisely three years old were included as research subjects.

### Oral health examination

The content of the oral examination encompasses dental caries, tooth loss, and treatment status. The examination focuses solely on the tooth’s crown. The oral examinations were conducted on a conventional chair under artificial lighting, combining visual inspection with probing using a flat mouth mirror and a 0.5 mm ball-ended CPI probe. Cotton swabs may be utilized to remove soft deposits when necessary. The diagnosis of dental caries adheres to the standards recommended by the WHO. The dmft index documents the experience of dental caries. A tooth is recorded as decayed (dt) if there is a clear cavity, enamel destruction, or detectable softening of the base or wall. A tooth is recorded as missing due to caries (mt) if it has been extracted because of dental caries. A tooth is recorded as filled (ft) if restorative material is observed. The prevalence of caries in deciduous teeth is typically represented by the average number of decayed teeth (dt), missing teeth due to caries (mt), and filled teeth (ft), known as deciduous caries, missing, and filled teeth (dmft), respectively.

### Questionnaire survey

The questionnaire used in our study was adapted from the WHO Oral Health Survey questionnaire [23] and revised by the Chinese Stomatological Association for national use. It was validated for repeatability in a pilot study conducted with a class of students [24] and was also utilised in the fourth national oral health epidemiological survey in 2015 [17]. The oral health questionnaire data were collected via face‒to-face interviews conducted by trained interviewers with parents or caregivers at the examination site. The questionnaire included information on oral disease-related risk factors, oral health knowledge, attitudes and practices, oral disease experience, and oral health service utilization. Two investigators were assigned to each survey team to complete all the oral health questionnaires at the surveillance sites.

### Statistical indicators

The research indicators include the prevalence of caries in primary teeth, the prevalence of SECC, the number of decayed teeth, the number of teeth filled or extracted due to caries (dmft), the significant caries index (SiC) score (average of the top one-third of dmft scores), and the SiC10 score (average of the top 10% of dmft scores). The diagnosis of dental caries follows WHO standards, and the prevalence of caries is quantified via the decayed, missing, and filled teeth index (dmft). A dmft score of ≥ 1 indicates the presence of caries, whereas a dmft score of ≥ 4 is classified as SECC.

### Quality control

The oral examiners included six dentists with professional qualifications and at least three years of clinical practice experience. Cohen’s kappa test was carried out with all examiners to verify the reliability of the diagnostic process. Two independent examiners, blinded from each other’s evaluations, assessed dental caries in a randomly selected sample of children based on WHO criteria. Statistical analysis was executed using SPSS, where a kappa coefficient exceeding 0.80 was regarded as indicative of excellent agreement. All examiners showed excellent inter-rater reliability, with kappa scores above 0.8. The recorders were medical professionals, specifically physicians or nurses with relevant dental clinical experience. During the oral health examination, 5% of the subjects were randomly selected for re-examination by an alternate examiner to ensure accuracy. Prior to departure from the examination site, the investigators conducted a thorough review of all questionnaire items. Any identified errors or omissions were promptly addressed and corrected. The questionnaires were signed, sealed, and submitted only after rigorous verification and confirmation.

### Data analysis

Two investigators independently entered the data via EpiData (EpiData Association, Odense, Denmark), and the two datasets were compared. Statistical analysis was conducted via SPSS version 26.0 (IBM Corp, Chicago, USA) and Stata version 18.0 (StataMP, College Station, TX, USA). Cohen’s kappa coefficient (k) was calculated to assess the consistency among the reviewers.

The prevalence of dental caries was determined on the basis of the dmft value of the study children. The response scores of parents regarding oral health knowledge and attitudes were as follows: 1 point for correct responses to oral health knowledge and positive responses to oral health attitudes, 0 points for incorrect responses to oral health knowledge and negative responses to oral health attitudes, and 0 points for all other responses. Chi-square tests were used to evaluate the differences in caries prevalence among the categorical groups. Since the dmft values were not normally distributed, nonparametric tests (Mann‒Whitney U test and Kruskal‒Wallis H test) were used to assess the differences in dmft distributions. The Mann‒Whitney U test and the Kruskal‒Wallis H test were used to compare the distributions of dmft scores among different groups. Logistic regression models were selected for variables (*p*<0.1) in the chi-square test. The standard negative binomial regression model, zero-inflated Poisson model, and zero-inflated negative binomial model were used to establish prediction models for the factors influencing the incidence of dental caries. The Vuong test and ZIP option test were used to determine the appropriate model. This study used the ZINB model to analyse the relationships between covariates and dmft scores. The zero-inflated part of the ZINB model revealed the relationships between covariates and caries (whether or not caries were present). The negative binomial part of the ZINB model was used to investigate the associations between positive caries experience (dmft > 0) and covariates. A backwards stepwise procedure was performed to remove the above nonsignificant variables (*p* ≥ 0.05), and all statistically significant variables (*p* < 0.05) were included in the final model. The significance level for all the statistical tests was set at *p* = 0.05.

## Results

Among the 1241 subjects (Table 1), 629 were boys (50.68%), and 612 were girls (49.32%), with a mean age of 3.5 ± 0.3 years. A total of 310 individuals exhibited dental caries, resulting in a caries prevalence rate of 25.00%. The mean dmft, SiC, and SiC10 indices were 0.93, 2.94, and 7.02, respectively. Notably, 117 children were diagnosed with SECC, yielding an SECC incidence of 9.43%. Specifically, 160 males exhibited dental caries, corresponding to a prevalence rate of 25.44%. In contrast, 150 females exhibited dental caries, with a prevalence rate of 24.51%. The SECC prevalence among girls was 8.5%, which was lower than the 10.33% reported in boys. However, no statistically significant differences were found in the overall prevalence of caries or SECC between males and females. Notably, the SiC10 value for boys (7.78) was significantly greater than that for girls (6.21) (*p* < 0.01). Additionally, the SiC10 value in suburban areas (8.02) was considerably greater than that in urban areas (6.46) (*p* < 0.01).

**Table 1:** The dental caries status of 3-year-old preschool children by gender and area in Shanghai, China (*n* = 1,241)

Variables		Sample Size(%)	Caries prevalence(%)	*p*-value (X^2^ test)	SECC prevalence(%)	*p*-value (X^2^ test)	Mean dmft ± SD	*p*-value (Mann-Whitney U)	Mean dt ± SD	*p*-value (Mann-Whitney U)	Mean ft ± SD	*p*-value (Mann-Whitney U)	SiC ± SD	*p*-value (Mann-Whitney U)	SiC10 ± SD	*p*-value (Mann-Whitney U)
	Total	1241(100%)	310(25.00%)		117(9.43%)		0.98 ± 2.40		0.93 ± 2.31		0.05 ± 0.42		2.94 ± 3.4		7.02 ± 3.52	
Gender	Boy	629(50.68%)	160(25.44%)	0.755	65(10.33%)	0.268	1.08 ± 2.62	0.553	1.05 ± 2.56	0.321	0.03 ± 0.25	0.076	3.24 ± 3.68	0.173	7.78 ± 3.60	0.002**
	Girl	612(49.32%)	150(24.51%)		52(8.50%)		0.88 ± 2.16		0.80 ± 2.02		0.08 ± 0.54		2.64 ± 3.07		6.21 ± 3.31	
Region	Urban	810(65.27%)	202(24.94%)	1.000	71(8.77%)	0.274	0.92 ± 2.26	0.796	0.85 ± 2.16	0.224	0.07 ± 0.47	0.488	2.76 ± 3.19	0.375	6.46 ± 3.49	0.003**
	Suburb	431(34.73%)	108(25.06%)		46(10.67%)		1.10 ± 2.66		1.06 ± 2.57		0.05 ± 0.42		3.29 ± 3.75		8.02 ± 3.46	

**p < 0.01

SiC Significant Caries Index, means of the top 1/3 of dmft scores

SiC10 Means of the top 10% of dmft scores

**Fig. 1:**
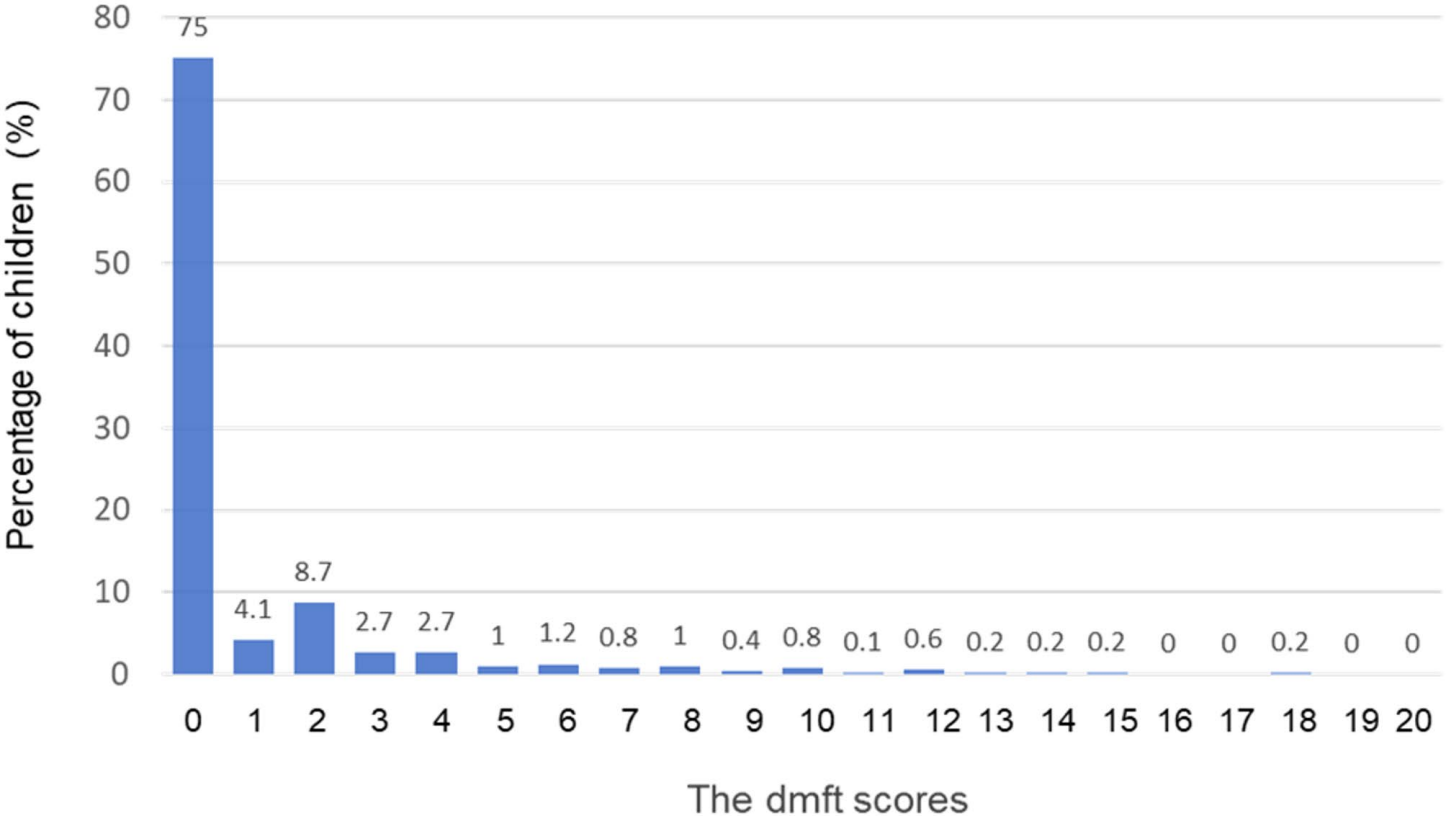
Proportion of dmft scores of 3-year-old children

**Fig. 2:**
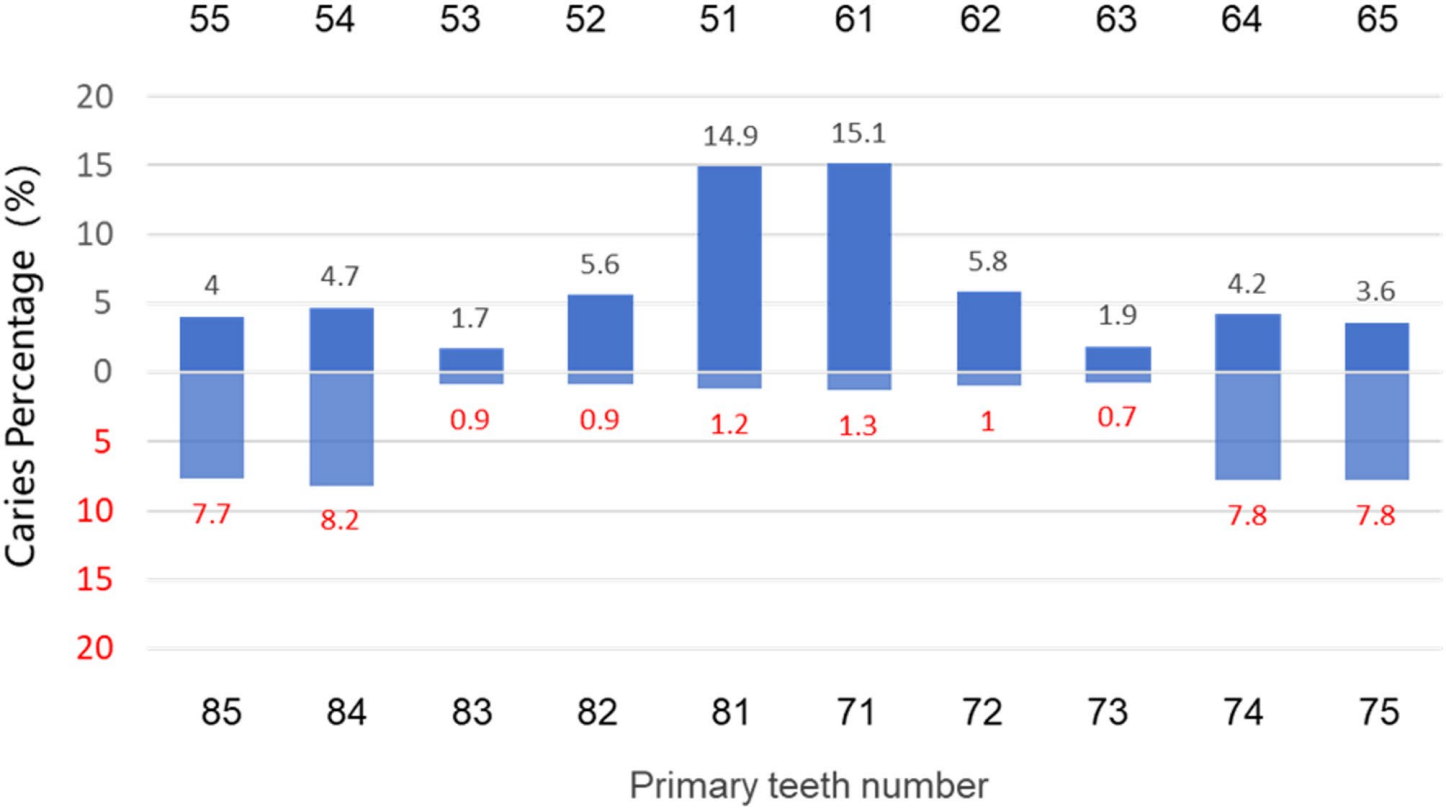
Distribution of the caries rate (dmft ≥ 1) of 3-year-old children by tooth position

Figure 1 illustrates the distribution of dmft scores among the examined children. The distribution exhibits positive skewness, with a skewness value of 3.5. Figure 2 presents the proportion of carious teeth at each tooth position. The X-axis denotes the positions of primary teeth in both the upper and lower dental arches, whereas the Y-axis represents the caries rate at each tooth position. The most common caries positions were as follows: maxillary central incisors (51 and 61) had the highest prevalence, followed by mandibular first molars (74 and 84), maxillary lateral incisors (52 and 62), maxillary first molars (54 and 64), mandibular second molars (75 and 85), mandibular lateral incisors (53 and 63), and mandibular central incisors (71 and 81). The maxillary central incisor had the highest caries rate (15.1%), followed by the mandibular bilateral primary molars (8.2%).

Table 2 summarizes the correlations between dental caries and various factors, including children’s oral health-related behaviors, mothers’ education levels, and parents’ perceptions of oral health. The prevalence of dental caries was significantly associated with the consumption of desserts (e.g., cake, biscuits, chocolate) (*p* < 0.05) and sugar-sweetened beverages (e.g., sweetened milk, yogurt, milk tea, soy milk) (*p* < 0.01) but not with other sweetened beverages (e.g., sugar-sweetened water, carbonated drinks, orange juice, apple juice, nonfreshly squeezed juices). Additionally, dental caries were significantly correlated with feeding frequency after bedtime brushing, age at which the child started to brush teeth, mother’s education level, dental experience, and parents’ self-evaluation of their children’s oral health status (*p* < 0.01).

**Table 2:** Dental caries prevalence and SECC prevalence in relation to dietary habits, oral hygiene behaviours, and caregiver oral health awareness and education

Variables	Caries prevalence *n*(%)	*p*-value	Mean dmft ± SD	*p*-value	SECC prevalence *n*(%)	*p*-value
**Dietary habits**						
Breastfeeding within 6 months of birth		0.219		0.138		0.397
Exclusively or Predominantly breastfed	200(26.53%)		1.10 ± 2.59		79(10.48%)	
Mixed fed(50/50)	65(21.45%)		0.67 ± 1.65		21(6.93%)	
Exclusively or Predominantly formula fed	44(24.18%)		1.01 ± 2.63		16(8.79%)	
Desserts		0.025*		0.023*		0.067
>1/week	623(26.88%)		1.07 ± 2.49		89(10.45%)	
≤ 1/week	303(20.89%)		0.81 ± 2.22		28(7.31%)	
Soft drink		0.125		0.074		0.071
>1/week	96(28.07%)		1.14 ± 2.45		74(12.57%)	
≤ 1/week	212(23.47%)		0.93 ± 2.39		43(8.82%)	
Sweet drink		<0.001**		<0.001***		0.001**
>1/week	157(30.02%)		1.30 ± 2.82		66(12.62%)	
≤ 1/week	149(21.02%)		0.75 ± 2.03		51(7.19%)	
Eating after bedtime brushing		<0.001**		<0.001***		<0.001**
>1/week	127(32.99%)		1.30 ± 2.62		52(13.51%)	
≤ 1/week	62(26.72%)		1.11 ± 2.66		21(9.05%)	
Never	121(19.52%)		0.74 ± 2.14		44(7.1%)	
**Oral hygiene behaviours**						
The age of brushing		0.001**		<0.001***		0.001**
<1 year old	55(19.86%)		0.68 ± 1.92		15(5.42%)	
1–2 years old	118(22.78%)		0.83 ± 2.16		41(7.92%)	
>2 years old	135(31.11%)		1.35 ± 2.86		60(13.82%)	
Frequency of brushing		0.067		0.040*		0.108
≥ 2/day	163(23.09%)		0.83 ± 2.11		57(8.07%)	
≤ 1/day	145(27.67%)		1.19 ± 2.76		59(11.26%)	
Use of floss with parents help		0.186		0.196		0.409
Every day	6(14.29%)		0.54 ± 1.82		3(7.14%)	
Not every day	61(23.46%)		0.83 ± 1.99		19(7.31%)	
Never	241(25.97%)		1.05 ± 2.54		95(10.24%)	
Dentist visit		<0.001**		<0.001***		<0.001**
Yes	121(33.61%)		1.50 ± 3.02		54(15%)	
No	188(21.61%)		0.78 ± 2.08		63(7.24%)	
**Caregiver oral health awareness and education**						
Mother's education level		0.003**		0.001**		0.003**
Junior college or below	80(30.50%)		1.38 ± 2.93		50(13.26%)	
Undergraduate or above	193(22.63%)		0.81 ± 2.12		66(7.74%)	
Evaluation of children's oral health status		<0.001**		<0.001***		<0.001**
Good	102(13.97%)		0.35 ± 1.09		19(2.6%)	
Moderate	134(34.99%)		1.27 ± 2.41		47(12.27%)	
Poor	70(63.64%)		4.27 ± 4.82		50(45.45%)	
Parents’ oral health knowledge		0.408		0.424		0.705
Score 0–2	6(25.00%)		1.08 ± 2.34		2(8.33%)	
Score 3–5	46(29.30%)		1.14 ± 2.60		18(1.14%)	
Score 6–8	258(25.34%)		0.96 ± 2.38		97(9.15%)	
Parents’ oral health attitude		0.419		0.462		0.357
Score 0–2	6(37.50%)		1.19 ± 1.68		3(18.75%)	
Score 3–4	14(26.92%)		1.06 ± 2.86		3(5.77%)	
Score 5–6	258(24.72%)		0.98 ± 2.39		111(9.46%)	

*p < 0.05, **p < 0.01

**Fig. 3:**
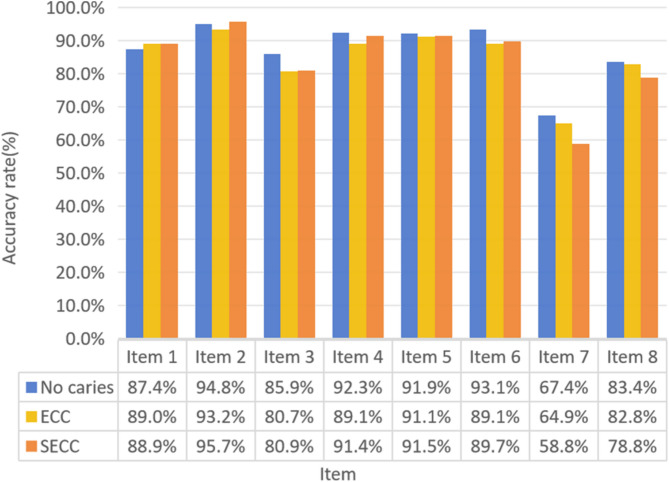
The parents’ oral health knowledge awareness rate. Item 1: Gingival bleeding during toothbrushing is abnormal; Item 2: Bacteria is associated with the onset of gingival inflammation; Item 3: Toothbrushing is effective in preventing gingival bleeding; Ltem 4: Bacteria can cause dental caries; and Ltem 5: Intake of sugars can cause dental caries.; ltem 6: Dental caries in deciduous dentition should not be neglected; ltem 7: Pit and fissure sealants can prevent dental caries in children.; ltem 8: Fluoride has a protective effect on teeth

**Fig. 4:**
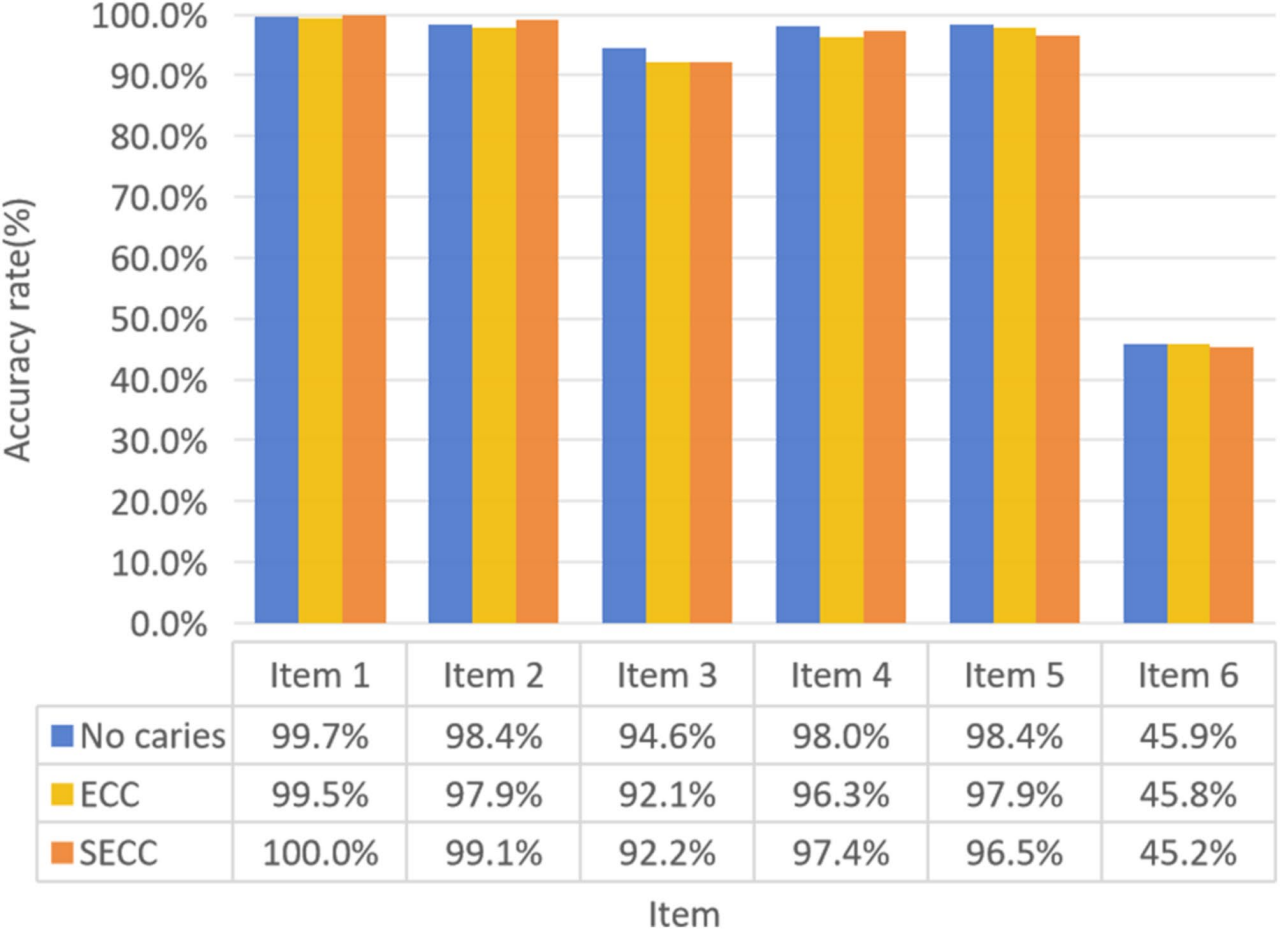
Parents’ oral health attitudes accuracy rate: ltem 1: Oral health is important; ltem 2: Regular oral health examinations are essential; ltem 3: The health of an individual’s teeth is not predetermined at birth; personal efforts significantly influence dental outcomes; ltem 4: Prevention of oral diseases primarily depends on oneself; ltem 5: Protecting children’s first permanent molars is crucial; ltem 6: The oral health status of mothers can have a significant impact on their children

Figures 3 and 4 provide a detailed analysis of the awareness rates regarding oral health knowledge and attitudes among parents of children in the three groups. A significant distinction was observed between the SECC and caries-free groups, particularly concerning the perception that pit and fissure sealing alone can prevent dental caries in children. Notably, the awareness rates for most aspects of oral health knowledge and attitudes exceeded 80%, with two exceptions: the effectiveness of pit and fissure sealing in preventing dental caries and the impact of mothers’ dental health on children’s teeth.

Logistic regression analysis of variables with *p* < 0.1 (Table 3) revealed that consuming sugar-sweetened beverages, eating after brushing one’s teeth before bedtime, and the age at which tooth brushing was initiated were significant risk factors associated with ECC. For SECC, logistic regression analysis further identified additional significant risk factors, including the mother’s education level. Specifically, participants who consumed sugar-sweetened beverages more than once a week had a greater risk of SECC than those who consumed them less frequently (OR = 1.65, 95% CI 1.07–2.55, *p* <0.05). Subjects who ate after brushing their teeth more than once a week had a greater risk of SECC than those who never engaged in this behavior did (OR = 1.62, 95% CI 1.01–2.59, *p* <0.05). Children who started brushing their teeth after two years had a greater risk of SECC than did those who began brushing before the age of one year (OR = 2.63, 95% CI 1.40–4.96, *p* <0.05). Furthermore, children whose mothers did not receive university education had a greater risk of SECC than those whose mothers had received university education did (OR = 1.61, 95% CI 1.07–2.43, *p* <0.05).

**Table 3:** Logistic regression analysis of factors associated with early childhood caries status

ECC	Variables		*p*-value	Odds ratio (OR)	95% CI	
					Lower	Upper
	Desserts					
		>1/week	0.244	1.21	0.88	1.67
		≤ 1/week (reference)				
	Soft drink					
		>1/week	0.561	0.91	0.66	1.25
		≤ 1/week (reference)				
	Sweet drink					
		>1/week	0.005**	0.91	0.66	1.25
		≤ 1/week (reference)				
	Eating after bedtime brushing					
		>1/week	<0.001**	1.77	1.29	2.43
		≤ 1/week	0.119	1.34	0.93	1.93
		Never (reference)				
	Frequency of brushing					
		≤ 1/day	0.576	1.09	0.81	1.45
		≥ 2/day (reference)				
	The age of brushing teeth(vs.<1 year old)					
		>2 years old	0.362	1.19	0.82	1.72
		1–2 years old	0.015*	1.63	1.10	2.41
		<1 year old (reference)				
	Mother’s education level(vs.undergraduate or above)					
		Junior college or below	0.081	1.29	0.97	1.71
		Undergraduate or above (reference)				

SECC	Variables		*p*-value	Odds ratio (OR)	95% CI	
					Lower	Upper
	Desserts					
		>1/week	0.358	1.26	0.77	2.08
		≤ 1/week (reference)				
	Soft drink					
		>1/week	0.610	1.13	0.71	1.79
		≤ 1/week (reference)				
	Sweet drink					
		>1/week	0.023*	1.65	1.07	2.55
		≤ 1/week (reference)				
	Eating after bedtime brushing					
		>1/week	0.045*	1.62	1.01	2.59
		≤ 1/week	0.746	1.10	0.62	1.93
		Never (reference)				
	Frequency of brushing					
		≤ 1/day	0.807	1.06	0.69	1.63
		≥ 2/day (reference)				
	The age of brushing teeth(vs.<1 year old)					
		>2 years old	0.003**	2.63	1.40	4.96
		1–2 years old	0.171	1.55	0.83	2.88
		<1 year old (reference)				
	Mother’s education level(vs.undergraduate or above)					
		Junior college or below	0.023*	1.61	1.07	2.43
		Undergraduate or above (reference)				

ECC: dmft ≥ 1; SECC: dmft ≥ 4

*p < 0.05, **p < 0.01

According to the negative binomial regression model (Table 4), both the age at the initiation of tooth brushing and the mother’s education level were significant predictors of the number of caries episodes. Children who initiated tooth brushing after the age of 2 years presented higher dmft scores (IRR = 1.58, 95% CI 1.13–2.20, *p* <0.05). Children whose mothers lacked a college education also had higher dmft scores (IRR = 1.47, 95% CI 1.15–1.87, *p* <0.05). In the logit component of the model, children who consumed sugar-sweetened beverages more than once a week were less likely to be free from dental caries (OR = 0.45, 95% CI 0.15–0.74, *p* <0.05), indicating an increased likelihood of developing dental caries. Similarly, children who ate snacks more than once a week after brushing their teeth were less likely to remain caries-free (OR = 0.67, 95% CI 0.34–1.01, *p* < 0.001), suggesting a greater risk of dental caries.

**Table 4:** Risk factors for dental caries in the surveyed children (ZINB regression moldel)

Risk factors	Odds ratio (OR)	95% CI	*p*-value
Zero-inflated portion (dmft = 0)			
Sweet drink			
>1/week	0.45	0.15–0.74	0.003**
≤ 1/week (reference)			
Eating after bedtime brushing			
>1/week	0.67	0.34–1.01	<0.001**
≤1/week	0.31	-0.10-0.72	0.143
Never (reference)			

Risk factors	Incidence rate ratio (IRR)	95% CI	*p*-value
Negative binomial portion (dmft > 0)			
The age of brushing			
>2 years old	1.58	1.13–2.20	0.007**
1–2 years old	1.20	0.86–1.69	0.287
<1 year old (reference)			
Mother's education level			
Junior college or below	1.47	1.15–1.87	0.002**
Undergraduate or above (reference)			

*p < 0.05, **p < 0.01

## Discussion

Compared with those in 2015, the prevalence of dental caries and the dmft index among 3-year-old children in Shanghai significantly declined in 2021. Specifically, the prevalence of caries decreased from 45.7 to 25.0%, whereas the dmft index decreased from 1.92 to 0.98 [18]. Notably, although the prevalence of caries remains slightly higher than that reported in Hong Kong (23%) [14], this discrepancy may be attributed to water fluoridation practices in Hong Kong [25, 26]. These findings suggest a marked improvement in the oral health status of 3-year-olds in Shanghai.

Fluoridation of drinking water has been carried out in many areas abroad, and community fluoridation of drinking water can reduce the incidence of severe early childhood caries [27]. However, mainland China has a high incidence of endemic fluorosis, affecting 28 provincial administrative regions and more than 70,000 villages [28]. Consequently, water fluoridation has not been universally adopted. Topical application of fluoride has been shown to effectively reduce the incidence of dental caries, irrespective of exposure to water fluoridation or other sources of fluoride [29]. Starting in 2020, Shanghai introduced topical fluoride treatments for children under three years of age. Oral examinations were integrated into routine neonatal health assessments to enhance mothers’ oral health education.

The SiC score for 3-year-old children in this survey was 2.94, which was significantly lower than the national epidemiological survey score of 6.04 reported in 2015 [17]. Compared with the dmft index, the SiC index indicates the urgency of oral treatment in children with multiple caries. Specifically, the greater the proportion of decayed, missing, or filled teeth (dmft) among the top 1/3 and top 10% of affected children is, the greater the severity of dental caries [30]. Among all the children with caries, 37.7% were diagnosed with severe SECC, underscoring the need for enhanced early screening and prevention of SECC. Identifying high-risk groups for caries is crucial for achieving timely prevention and treatment.

The caries status of the left and right sides was largely symmetrical. The maxillary central incisors and mandibular primary molars presented a greater prevalence of caries than the other tooth positions did, which aligns with the findings of previous studies [14, 17, 31]. Emphasis should be placed on preventing and treating caries in maxillary primary anterior teeth and mandibular primary molars. Caries in upper anterior teeth represent an atypical pattern of caries onset in ECC [32]. The decay of maxillary primary anterior teeth may be associated with poor feeding habits, early eruption, frequent posteruption feeding, nocturnal feeding, and inadequate oral hygiene after meals. Longitudinal studies of 9-month-old infants at 12 and 18 months of age revealed that caries developed on the labial surface of maxillary incisors within six months of eruption and progressively worsened over time [33]. Therefore, prompt medical intervention and the application of fluoride following the eruption of maxillary central incisors are crucial for preventing the progression of primary tooth caries.

Sugar consumption has been strongly linked to the incidence of dental caries, with the frequency of intake being a critical factor. This study revealed a significant association between the frequency of desserts and sweetened beverages and the prevalence of caries and dmft scores. However, logistic regression analysis revealed that only the frequency of sugar-sweetened beverage consumption was significantly associated with the prevalence of caries and the SECC. Numerous studies have documented a direct dose‒response relationship between sugar intake and caries development, which is supported by clear biological evidence indicating that cariogenic bacteria flourish in fermentable carbohydrates such as sucrose, fructose, and glucose [34, 35]. Prolonged and frequent exposure of teeth to these fermentable carbohydrates, particularly sugars, creates an acidic plaque environment conducive to enamel demineralization, thereby initiating the process of dental caries [36]. A 2021 study of 3–5-year-old children in Victoria, Australia, revealed that those who consumed soft drinks once or more per week presented a 1.66-fold increased risk of developing dental caries compared with those who never or rarely consumed such beverages [37]. However, the study revealed no significant associations between soft drink consumption and caries or SECC. This lack of association may be attributed to the relatively low proportion of children consuming soft drinks at least once a week (27.6%), as opposed to the higher percentages for sweet foods (68.7%) and sweetened beverages (42.1%). Reducing early exposure to sugar and decreasing the frequency of sugar intake may help prevent the onset of ECC. Consequently, future oral health education initiatives should focus on enhancing parental awareness regarding the frequency of their children’s consumption of sugary items to control the incidence of dental caries better.

Brushing is an effective method for removing plaque and preventing dental caries. The frequency of eating after teeth are brushed before bedtime significantly correlates with the severity of caries in primary teeth, which is consistent with prior studies [17, 30]. Consuming food after bedtime brushing significantly diminishes the efficacy of dental hygiene, particularly when it involves sticky, sugar-rich items such as milk and confectionery. These substances tend to leave residues on the teeth, thereby nourishing the cariogenic bacteria within the oral cavity. The age at which children begin brushing their teeth is closely associated with the severity of deciduous tooth caries. Research has shown that children who start brushing later are more prone to developing primary teeth caries [30]. Our study revealed that children who began brushing at 1–2 years of age were more susceptible to caries, whereas those who started brushing after 2 years had a greater risk of SECC than those who began brushing before 1 year of age. Negative binomial regression analysis also indicated that the age at which children start brushing is related to the severity of caries; the later they start, the greater the number of carious teeth. Therefore, we recommend initiating tooth brushing no later than 1 year of age, which aligns with the findings of Wong et al. [38]. The correlation between tooth brushing frequency and dental caries has been well established in numerous studies [39–41]. In this study, only 56.9% of the children reported brushing their teeth more than twice daily. A significant chi-square test difference was observed between tooth brushing frequency and the mean dmft score; however, no significant differences were found concerning the caries rate and SECC. Consistent with prior research, a lower frequency of tooth brushing was associated with a higher mean dmft score, reflecting a gradual increase in dental caries as the brushing frequency decreased [42, 43]. While this study focused on brushing frequency, it is important to note that brushing duration and technique may be even more critical factors in assessing the effectiveness of dental plaque removal. Future research should refine these variables to better understand the relationship between brushing habits and dental caries.

This study revealed a significant correlation between mothers’ education level and the caries status of their children. Specifically, higher maternal education levels were associated with a lower incidence and severity of dental caries in children, findings that align with previous research [17, 40, 44, 45]. This relationship may be attributed to mothers with higher education levels possessing greater awareness of oral health knowledge and demonstrating increased concern for their children’s oral health. The mothers of the children who participated in the oral examination in Shanghai demonstrated a notably high level of educational attainment, with 68.7% holding a college degree or higher. These mothers firmly grasp oral health knowledge and maintain a positive attitude. A recent systematic review tentatively identified low maternal education level as a risk factor for future caries development [46]. As primary decision-makers, parents play a crucial role in children’s oral health. Positive parental attitudes and comprehensive knowledge of oral health have significantly improved children’s oral quality of life [47–49]. In this study, caregivers’ evaluations of children’s oral health status were positively correlated with the prevalence of caries, suggesting a more accurate understanding of children’s oral health among caregivers.

Currently, a prevalent trend among primary school students in China is that more than half of them undergo dental examinations for treatment. In 2020, Chen et al. reported a positive correlation between dental visits within the past 12 months and the prevalence of caries in Wuhan [24]. Older children are more likely to visit dentists [37]. Children aged three years cannot generally cooperate with outpatient treatments and examinations, and parents typically only seek dental care when their child experiences significant pain, primarily for pain relief. Consequently, the prevention and early detection of deciduous dental caries in young children are crucial. Early preventive measures, such as enhanced screening and monitoring of high-risk children, regular oral examinations for preschool-aged children, pit and fissure sealants, and topical fluoride treatments, should be implemented to minimize the incidence of caries and SECC in primary teeth.

The reduced caries prevalence and dmft index among 3-year-old children in Shanghai may be attributed to effective preventive measures and heightened parental awareness of oral health. Nonetheless, continuous monitoring of the oral health status of this age group, particularly those at high risk for caries, remains essential. Promoting effective oral health interventions is crucial to maintaining and enhancing their oral health. This study provides an update on the oral health status of 3-year-old children in Shanghai; however, it has certain limitations. Initially, it is crucial to recognise that the inherent nature of a cross-sectional study precludes the establishment of causality. Parents or caregivers may exhibit response bias when answering questions, potentially providing answers that they believe are socially desirable rather than truthful. Secondly, owing to concerns regarding participant privacy and the sensitive nature of income and occupation data, we encountered significant difficulties in collecting accurate and reliable information on these variables. A substantial proportion of participants exhibited reluctance to disclose detailed income specifics, resulting in collected data that were frequently incomplete or of questionable accuracy. Consequently, critical confounding variables—such as family socioeconomic status, which may influence study outcomes—were not comprehensively accounted for in our analysis. Future prospective studies are necessary to investigate the impact of oral hygiene measures further.

## Conclusions

The prevalence of dental caries among 3-year-old children in Shanghai is low, yet the rate of SECC remains a concern. SECC is associated with factors including the frequency of sugar-sweetened beverage intake, post-toothbrushing eating habits, age of toothbrushing initiation, and mother’s education level. Developing tailored and effective preventive strategies for this population is crucial. These strategies could involve reducing sugar-sweetened beverage consumption, eliminating post-brushing food intake before bedtime, implementing oral health education programmes for parents and caregivers, and promoting early and consistent toothbrushing habits.

## Data Availability

The datasets generated and/or analysed in this study are not publicly accessible because they provide signed informed consent and ethical requirements. However, they can be made available to the corresponding author upon reasonable request.

## Abbreviations

CI: Confdence intervals
dmft: Number of dental caries, extractions, or fillings in primary teeth due to dental caries
ECC: Early childhood caries
GDP: Gross domestic product
IRR: Incidence rate ratio
OR: Odds ratio
SECC: Severe early childhood caries
SiC10: Means of the top 10% of dmft scores
SiC: Significant Caries Index, means of the top 1/3 of dmft scores
WHO: World Health Organization
ZINB: Zero‒inflated negative binomial

## References

[1] Su H, Yang R, Deng Q, Qian W, Yu J. Deciduous dental caries status and associated risk factors among preschool children in Xuhui district of Shanghai, China. BMC Oral Health. 2018;18(1):111. 29921269 10.1186/s12903-018-0565-8 PMC6009057

[2] Anil S, Anand PS. Early childhood caries: prevalence, risk factors, and prevention. Front Pediatr. 2017;5:157. 28770188 10.3389/fped.2017.00157 PMC5514393

[3] Galán CA, Shaw DS, Dishion TJ, Wilson MN. Neighborhood deprivation during early childhood and conduct problems in middle childhood: mediation by aggressive response generation. J Abnorm Child Psych. 2017;45(5):935–46. 10.1007/s10802-016-0209-x PMC5927582 27696324

[4] Kassebaum NJ, Bernabé E, Dahiya M, Bhandari B, Murray CJ, Marcenes W. Global burden of untreated caries: a systematic review and metaregression. J Dent Res. 2015;94(5):650–8. 25740856 10.1177/0022034515573272

[5] World Health Organization. Global oral health status report (2022). World Health Organization. 2022. https://www.who.int/news-room/fact-sheets/detail/oral-health. Accessed 6 Nov 2024.

[6] Policy on Early Childhood Caries (ECC). Classifications, consequences, and preventive strategies. Pediatr Dent. 2018;40(6):60–2. 32074852

[7] Tinanoff N, Baez RJ, Diaz Guillory C, Donly KJ, Feldens CA, McGrath C, Phantumvanit P, Pitts NB, Seow WK, Sharkov N, Songpaisan Y, Twetman S. Early childhood caries epidemiology, aetiology, risk assessment, societal burden, management, education, and policy: global perspective. Int J Paediatr Dent. 2019;29(3):238–48. 31099128 10.1111/ipd.12484

[8] Uribe SE, Innes N, Maldupa I. The global prevalence of early childhood caries: a systematic review with meta-analysis using the WHO diagnostic criteria. Int J Paediatr Dent. 2021;31(6):817–30. 33735529 10.1111/ipd.12783

[9] Kane SF. The effects of oral health on systemic health. Gen Dent. 2017;65(6):30–4. 29099363

[10] Naidu R, Nunn J, Donnelly Swift E. Oral health-related quality of life and early childhood caries among preschool children in Trinidad. BMC Oral Health. 2016;16(1):128. 27923355 10.1186/s12903-016-0324-7 PMC5142136

[11] Alanzi A, Husain F, Husain H, Hanif A, Baskaradoss JK. Does the severity of untreated dental caries of preschool children influence the oral health-related quality of life? BMC Oral Health. 2023;23(1):552. 37563589 10.1186/s12903-023-03274-7 PMC10416462

[12] Şengül F, Urvasızoğlu G, Derelioǧlu S, Seddik T, Çelikel P, Baş A. Early childhood caries in 4- to 5-Year-old children in Erzurum, Turkey. Front Public Health. 2021;9:725501. 34900887 10.3389/fpubh.2021.725501 PMC8661086

[13] Masood M, Yusof N, Hassan MI, Jaafar N. Assessment of dental caries predictors in 6-year-old school children - results from 5-year retrospective cohort study. BMC Public Health. 2012;12:989. 23158416 10.1186/1471-2458-12-989 PMC3524020

[14] Sun IG, Duangthip D, Yan IG, Zheng FM, Lo ECM, Chu CH. Caries incidence and its associated factors in Hong Kong kindergarten children. Int Dent J. 2024;75(2):761–6. 38945801 10.1016/j.identj.2024.05.015 PMC11976464

[15] Isaksson H, Alm A, Koch G, Birkhed D, Wendt LK. Caries prevalence in Swedish 20-year-olds in relation to their previous caries experience. Caries Res. 2013;47(3):234–42. 23328627 10.1159/000346131

[16] Gu ZW, Zhang SS, Zhang RJ, Tang H, Sun XY, Liu XN, Zheng SG. Prevalence of caries in Mainland China: evidence from 1980 to 2018: a systematic review and meta-analysis. Chin J Dent Res. 2019;22(4):251–63. 31859285 10.3290/j.cjdr.a43736

[17] Du MQ, Li Z, Jiang H, Wang X, Feng XP, Hu Y, Lin HC, Wan B, Si Y, Wang CX, Zheng SG, Liu XN, Rong WS, Wang WJ, Tai BJ. Dental caries status and its associated factors among 3- to 5-year-old children in China: a national survey. Chin J Dent Res. 2018;21(3):167–79. 30255168 10.3290/j.cjdr.a41076

[18] Wang HN, Wang Y, Zhang H, Mao YM, Dong H, Hua M, Jiang YW, Zhang Y. Early childhood caries and its related risk factors in 1 296 children aged 3 to 5 years old in Shanghai. Shanghai Kou Qiang Yi Xue. 2020;29(2):174–8. [in Chinese]. 32626881

[19] Chen KJ, Gao SS, Duangthip D, Lo ECM, Chu CH. Early childhood caries and oral health care of Hong Kong preschool children. Clin Cosmet Investig Dent. 2019;11:27–35. 30697084 10.2147/CCIDE.S190993 PMC6340357

[20] Arora A, Manohar N, John JR. Factors associated with dental caries in primary dentition in a non-fluoridated rural community of New South Wales, Australia. Int J Environ Res Public Health. 2017;14(12). 10.3390/ijerph14121444 PMC5750863 29168780

[21] Kim Seow W. Environmental, maternal, and child factors which contribute to early childhood caries: a unifying conceptual model. Int J Paediatr Dent. 2012;22(3):157–68. 21972925 10.1111/j.1365-263X.2011.01186.x

[22] Abanto J, Diaz Cárdenas S, Veloso Duran A, Garza M, Reis Brigato V, Guinot F. Association between socioeconomic factors, attitudes and beliefs regarding the primary dentition and caries in children aged 1–5 years of Brazilian and Colombian parents. Eur J Paediatr Dent. 2024;25(4):258–65. 38888576 10.23804/ejpd.2024.2102

[23] Petersen PE, Baez RJ, World Health O. Oral health surveys: basic methods. 5th ed. edn. Geneva: World Health Organization; 2013.

[24] Chen L, Hong J, Xiong D, Zhang L, Li Y, Huang S, Hua F. Are parents’ education levels associated with either their oral health knowledge or their children’s oral health behaviors? A survey of 8446 families in Wuhan. BMC Oral Health. 2020;20(1):203. 32652985 10.1186/s12903-020-01186-4 PMC7353758

[25] Chen J, Duangthip D, Gao SS, Huang F, Anthonappa R, Oliveira BH, Turton B, Durward C, El Tantawi M, Attia D, Heima M, Muthu MS, Maharani DA, Folayan MO, Phantumvanit P, Sitthisettapong T, Innes N, Crystal YO, Ramos-Gomez F, Medina AC, Lo ECM, Chu CH. Oral health policies to tackle the burden of early childhood caries: a review of 14 countries/regions. Front Oral Health. 2021;2:670154. 35048013 10.3389/froh.2021.670154 PMC8757786

[26] Zheng FM, Yan IG, Sun IG, Duangthip D, Lo ECM, Chu CH. Early childhood caries and dental public health programmes in Hong Kong. Int Dent J. 2024;74(1):35–41. 37839956 10.1016/j.identj.2023.08.001 PMC10829355

[27] Lee HH, Faundez L, LoSasso AT. A cross-sectional analysis of community water fluoridation and prevalence of pediatric dental surgery among medicaid enrollees. JAMA Netw Open. 2020;3(8):e205882. 32785633 10.1001/jamanetworkopen.2020.5882 PMC7424407

[28] Zhao L, Li Z, Li M, Sun H, Wei W, Gao L, Zhao Q, Liu Y, Ji X, Li C, Wang J, Gao Y, Pei J. Spatial-temporal analysis of drinking water type of endemic fluorosis - China, 2009–2022. China CDC Wkly. 2024;6(2):25–9. 38250699 10.46234/ccdcw2024.006 PMC10797302

[29] Marinho VC, Higgins JP, Logan S, Sheiham A. Topical fluoride (toothpastes, mouthrinses, gels or varnishes) for preventing dental caries in children and adolescents. Cochrane Database Syst Rev. 2003;(4):CD002782. 10.1002/14651858.CD002782 PMC6999805 14583954

[30] Liu Y, Zhu J, Zhang H, Jiang Y, Wang H, Yu J, Da D, Chen Q, Su H, Wu Z, Shi H, You J, Zeng X, Zhang Y. Dental caries status and related factors among 5-year-old children in Shanghai. BMC Oral Health. 2024;24(1):459. 38627729 10.1186/s12903-024-04185-x PMC11020175

[31] Zeng L, Zeng Y, Zhou Y, Wen J, Wan L, Ou X, Zhou X. Diet and lifestyle habits associated with caries in deciduous teeth among 3- to 5-year-old preschool children in Jiangxi province, China. BMC Oral Health. 2018;18(1):224. 30572852 10.1186/s12903-018-0686-0 PMC6302433

[32] Wyne AH. Early childhood caries: nomenclature and case definition. Community Dent Oral. 1999;27(5):313–5. 10.1111/j.1600-0528.1999.tb02026.x 10503790

[33] Chanpum P, Duangthip D, Trairatvorakul C, Songsiripradubboon S. Early childhood caries and its associated factors among 9- to 18-month old exclusively breastfed children in Thailand: a Cross-Sectional study. Int J Environ Res Public Health. 2020;17(9). 10.3390/ijerph17093194 PMC7246726 32375351

[34] Colak H, Dülgergil CT, Dalli M, Hamidi MM. Early childhood caries update: a review of causes, diagnoses, and treatments. J Nat Sci Biol Med. 2013;4(1):29–38. 23633832 10.4103/0976-9668.107257 PMC3633299

[35] Sheiham A, James WP. A reappraisal of the quantitative relationship between sugar intake and dental caries: the need for new criteria for developing goals for sugar intake. BMC Public Health. 2014;14:863. 25228012 10.1186/1471-2458-14-863 PMC4168053

[36] Nakai Y, Mori-Suzuki Y. Impact of dietary patterns on plaque acidogenicity and dental caries in early childhood: a retrospective analysis in Japan. Int J Environ Res Public Health. 2022;19(12). 10.3390/ijerph19127245 PMC9223666 35742494

[37] Graesser H, Sore R, Rogers J, Cole D, Hegde S. Early childhood caries in Victorian preschoolers: a cross-sectional study. Int Dent J. 2022;72(3):381–91. 34247833 10.1016/j.identj.2021.05.013 PMC9275113

[38] Wong MC, Lu HX, Lo EC. Caries increment over 2 years in preschool children: a life course approach. Int J Paediatr Dent. 2012;22(2):77–84. 21771124 10.1111/j.1365-263X.2011.01159.x

[39] Liu J, Zhang SS, Zheng SG, Xu T, Si Y. Oral health status and oral health care model in China. Chin J Dent Res. 2016;19(4):207–15. 27995225 10.3290/j.cjdr.a37145

[40] Boonyawong M, Auychai P, Duangthip D. Risk factors of dental caries in preschool children in Thailand: a cross-sectional study. Healthc (Basel). 2022;10(5). 10.3390/healthcare10050794 PMC9140486 35627931

[41] Liu M, Song Q, Xu X, Lai G. Early childhood caries prevalence and associated factors among preschoolers aged 3–5 years in Xiangyun, China: a cross-sectional study. Front Public Health. 2022;10:959125. 36052000 10.3389/fpubh.2022.959125 PMC9424677

[42] Kumar S, Tadakamadla J, Johnson NW. Effect of toothbrushing frequency on incidence and increment of dental caries: a systematic review and meta-analysis. J Dent Res. 2016;95(11):1230–6. 27334438 10.1177/0022034516655315

[43] Ghasemianpour M, Bakhshandeh S, Shirvani A, Emadi N, Samadzadeh H, Moosavi Fatemi N, Ghasemian A. Dental caries experience and socioeconomic status among Iranian children: a multilevel analysis. BMC Public Health. 2019;19(1):1569. 31775741 10.1186/s12889-019-7693-1 PMC6880421

[44] Abbass MMS, Mahmoud SA, El Moshy S, Rady D, AbuBakr N, Radwan IA, Ahmed A, Abdou A, Al Jawaldeh A. The prevalence of dental caries among Egyptian children and adolescences and its association with age, socioeconomic status, dietary habits and other risk factors. A cross-sectional study. F1000Res. 2019;8:8. 30854195 10.12688/f1000research.17047.1 PMC6396843

[45] Chen Z, Zhu J, Zhao J, Sun Z, Zhu B, Lu H, Zheng Y. Dental caries status and its associated factors among schoolchildren aged 6–8 years in hangzhou, China: a cross-sectional study. BMC Oral Health. 2023;23(1):94. 36788543 10.1186/s12903-023-02795-5 PMC9926711

[46] Lam PPY, Chua H, Ekambaram M, Lo ECM, Yiu CKY. Does early childhood caries increase caries development among school children and adolescents?? A systematic review and Meta-Analysis. Int J Environ Res Public Health. 2022;19:20. 10.3390/ijerph192013459 PMC9603429 36294037

[47] Liu SM, Xin YM, Wang F, Lin PC, Huang HL. Parental health belief model constructs associated with oral health behaviors, dental caries, and quality of life among preschool children in China: a cross-sectional study. BMC Oral Health. 2024;24(1):1497. 39696114 10.1186/s12903-024-05290-7 PMC11657512

[48] Vann WF, Lee JY, Baker D, Divaris K. Oral health literacy among female caregivers: impact on oral health outcomes in early childhood. J Dent Res. 2010;89(12):1395–400. 20924067 10.1177/0022034510379601 PMC3123718

[49] Bozorgmehr E, Hajizamani A, Malek Mohammadi T. Oral health behavior of parents as a predictor of oral health status of their children. ISRN Dent. 2013;2013:741783. 10.1155/2013/741783 PMC3664493 23738088

